# Ground States for Metals from Converged Coupled Cluster Calculations

**DOI:** 10.1021/acs.jpclett.4c03134

**Published:** 2024-12-18

**Authors:** Tobias Schäfer

**Affiliations:** Institute for Theoretical Physics, TU Wien, Wiedner Hauptstraße 8-10/136, A-1040 Vienna, Austria

## Abstract

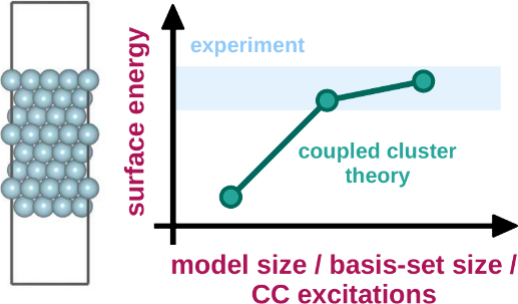

Many-electron correlation methods offer a systematic approach to predicting material properties with high precision. However, practically attaining accurate ground-state properties for bulk metals presents significant challenges. In this work, we propose a novel scheme to reach the thermodynamic limit of the total ground-state energy of metals using coupled cluster theory. We demonstrate that the coupling between long-range and short-range contributions to the correlation energy is sufficiently weak, enabling us to restrict long-range contributions to low-energy excitations in a controllable way. Leveraging this insight, we calculated the surface energy of aluminum and platinum (111), providing numerical evidence that coupled cluster theory is well-suited for modeling metallic materials, particularly in surface science. Notably, our results exhibit convergence with respect to finite-size effects, basis-set size, and coupled cluster expansion, yielding excellent agreement with experimental data. This paves the way for more efficient coupled cluster calculations for large systems and a broader utilization of theory in realistic metallic models of materials.

Accurately predicting quantum mechanical ground-state properties is fundamental to materials modeling and requires the most advanced computational methods. Systematically improvable many-electron correlation methods play a pivotal role, with the family of coupled cluster (CC) methods standing out as a prominent and widely recognized approach.^1−4^ Within CC methods, the true many-electron wave function |ψ⟩ is approximated through excitations of a mean-field Slater determinant |ϕ⟩—mostly from Hartree–Fock (HF) theory—with accuracy systematically enhanced through consideration of higher-order excitations *T* = *T*_1_ + *T*_2_ + ... by means of an exponential ansatz |ψ⟩ = e^*T*^|ϕ⟩.

In materials science, the accuracy is often considered in the notion of a hypothetical three-dimensional parameter space encompassing the employed simulation cell size, the one-electron basis-set size, and the order of excitation operators in the CC method. Given a desired accuracy necessitates convergence of all three parameter axes, encompassing the thermodynamic limit (TDL), the complete basis set limit (CBS), and the limit of excitation orders, respectively. Due to the steep scaling of the computational cost along all three axes, achieving converged coupled cluster calculations is often practically infeasible for extended systems like solids.^4,5^ For metals, in particular, the goal of materials modeling from converged CC results often remains out of reach.^6,7^ The limited number of fully converged CC results leaves gaps in our understanding of the theory’s performance for materials.

In this work, we present a novel finite-size correction scheme to reach the TDL of CC ground-state energies for real metals. This scheme, combined with recent methodological advancements, forms a robust and massively parallelized computational framework for calculating converged CC ground-state properties across all three hypothetical parameters. We apply this framework to determine the surface energies of aluminum and platinum in the (111) termination.

Surface properties are crucial in various research fields, including heterogeneous catalysis, energy storage, and corrosion, as materials primarily interact with their environment at their surfaces. While density functional theory (DFT) is undeniably the leading method used in materials modeling, existing approximations of the exchange-correlation functional often fail to reliably predict surface energies.^8^ More advanced methods like the random phase approximation (RPA) show improved performance over DFT for metal surface energies^9−11^ but suffer from known issues, introducing uncertainties in the results. Notably, RPA suffers from unphysical self-correlation, which only cancels out for energy differences of similar electronic structures.^12−15^ A study systematically approaching the exact solution of the many-electron Schrödinger equation for surface models is still lacking.

*Methods.* Our approach to systematically converge CC theory is a synthesis of our novel finite-size correction scheme to reach the TDL with several recently published methodological advancements. Starting from a plane-wave basis representation of the HF orbitals from the Vienna Ab-initio Simulation Package (VASP),^16−18^ we efficiently reach the CBS through a transformation to a more compact natural orbital basis^19,20^ in combination with a highly effective focal point correction scheme.^21^ An efficient solution to the long-standing problem of accurately capturing three-electron correlation effects in metals without running into an infrared catastrophe^22,23^—as is the case in CCSD(T) using the perturbative triples treatment (T)^24^—has recently been proposed by us.^25^ In this new approach, denoted as CCSD(cT), significant three-electron screening effects are accounted for, preventing the infrared catastrophe and providing an accurate estimate for coupled cluster calculations with single, double, and triple excitations.^26^ Using our massively parallelized cc4s code,^27^ we show that for the surface energy of aluminum and platinum (111) convergence of the excitation orders is reached using CCSD(cT). Furthermore, two points are crucial for the successful calculation of the ground states of metals in this work. First, we employ a Monte Carlo integration technique to sample the Brillouin zone, which allows us to converge the HF and CC energy of a finite simulation cell without relying on high-symmetry points.^28^ This so-called twist-averaging technique is important as it prevents degenerate HF energies and ensures purely integer orbital occupation numbers.^6^ Additionally, we utilize a recently published sampling technique for the reciprocal Coulomb potential, which enables converged HF and CC ground-state energies in anisotropic simulation cells,^29^ as necessary for modeling surface slabs. In the following, we introduce our novel finite-size correction scheme.

*Achieving the TDL of Real Metals.* The correlation energy per unit, such as an atom or unit cell, of a periodic system can be expressed as
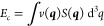
1where *v*(***q***) = 4π/***q***^2^ is the Fourier transform of the Coulomb potential 1/|***r***| and we call *S*(***q***) the transition structure factor^30^ per unit. The transition structure factor can be interpreted as the Fourier transform of the transition pair correlation function,^31^ encompassing the physical information about the many-electron correlations in the system, and is determined by the many-electron Schrödinger equation. More formally, it can also be considered as the partial derivative of the correlation energy *E*_*c*_ with respect to the reciprocal potential *v*(***q***). In this work *S*(***q***) serves as the main quantity to study different correlation lengths as long-range and short-range correlation effects are decoded in small and large magnitudes of the transition vectors ***q***, respectively.^32^ This is a consequence of the fact that we consider the Fourier transformations of real space quantities, i.e., ***q*** is the conjugate variable of ***r***. In plane wave and real-space grid based approaches, in particular, ***q***-dependent quantities are inherently accessible. In any finite-cell simulation, the reciprocal transition vectors ***q***, also known as momentum transfer vectors, can be decomposed into the sum of a reciprocal lattice vector and a difference vector of two grid points sampling the first Brillouin zone (BZ).^32^ Hence, a finite grid of ***q*** vectors is defined by the sampling grid of the BZ, the unit cell, and a cutoff parameter which specifies the largest |***q***| to be considered. In a practical computation, the integral of eq 1 turns into a sum over this grid. We note that the singularity at ***q*** = 0, although integrable, requires careful handling.^29,33^

In coupled cluster theory the structure factor *S*(***q***) is accessible through the double excitation amplitudes *t*_*ij*_^*ab*^ as
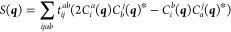
2where *C*_*i*_^*a*^(***q***) are the Fourier transforms of the overlap densities φ_i_^*^(***r***)φ_*a*_(***r***) of the occupied and unoccupied (virtual) mean-field orbitals *i* and *a*, respectively. The double excitation amplitudes *t*_*ij*_^*ab*^ are solutions of the CC amplitude equations which are derived from the CC ansatz mentioned in the introduction and the time-independent Schrödinger equation. Considering coupled cluster with single and double particle–hole excitation operators (CCSD) the amplitude equations are basically self-consistent equations, consisting of contractions of the Coulomb tensor *v*_*ij*_^*ab*^, the amplitudes themselves, as well as HF orbital energy differences Δ_*ab*_^*ij*^ = ε_*i*_ + ε_*j*_ – ε_*a*_ – ε_*b*_,
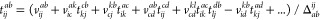
3Summation over repeated indices is assumed. We only show selected terms of the CCSD equations (for the full set of terms we refer to ref (34)) to illustrate that *S*(***q***) explicitly depends on both the ***q*** grid and the amplitudes, while the amplitudes implicitly depend on the ***q*** grid through the Coulomb integrals. Under periodic boundary conditions, the Coulomb integrals *v*_*ij*_^*ab*^ can be calculated as a sum over the grid,
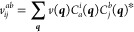
4a formulation naturally accessible in a plane wave basis. In practice, the sum over ***q*** includes a weighting factor that depends on the chosen integration grid, but this has been omitted here for brevity.

In this context, the finite-size error in the correlation energy—i.e., deviations of the correlation energy from its value in the thermodynamic limit—can be attributed to the grid’s coarseness leading to missing information near ***q*** = 0. This finite-size error diminishes as the sampling of the BZ becomes infinitely dense or, equivalently, as the simulation cell becomes infinitely large.

Few strategies exist for correcting finite-size errors,^33,35,36^ with cell-size extrapolation techniques^37^ being among the most commonly used. Alternatively, the structure factor offers another method to estimate these corrections. Liao et al.^32^ introduced a structure factor interpolation scheme. This method approximates the limit of an infinitely dense ***q*** grid (the TDL) by using a tricubic interpolation technique based on *S*(***q***) on the finite grid.

Inspired by the structure factor based approach, we employ a multiscale approach here, which aims to approximate the small ***q*** contributions missing in the grid. To this end, we probe the coupling strength of small and large ***q*** contributions. In other words, can *S*(***q***) be accurately calculated for a small ***q*** while neglecting larger ***q***? One way to scrutinize this is by solving the CCSD equations using a potential which smoothly damps large transition momentum vectors, like . In real space, this corresponds to a long-range (LR) potential using the error function 1/|***r***| → erf(μ|***r***|)/|***r***|. We use the prefix LR to indicate solutions of the CC equations employing a long-range potential, such as LRCCSD for long-range CCSD. For simplicity, we chose a fixed parameter setting of μ = 1.0 Å^–1^ for the error function throughout this work. This setting defines a potential that meets the Coulomb potential for distances larger than *r* > 2/μ = 2 Å, since erf(2) > 0.99. In passing, we note that the Wigner-Seitz radius—commonly used to characterize electron densities in simple metals—is typically between 1 and 3 Å in models such as the uniform electron gas, where it is used to approximate real metals.^38,39^

Figure 1 shows the structure factor *S*(***q***) at the level of the CCSD for bulk aluminum in the fcc structure. For the calculation a 3 × 3 × 3 supercell of the conventional unit cell was employed, resulting in a finite-size model containing 108 atoms, as visualized in the Supporting Information (SI).^40^ The smallest finite transition momentum vector is determined by this choice. The difference between the structure factors of the CCSD and the LRCCSD calculation approaches zero for small |***q***|. Hence, both structure factors describe similar long-range effects in the correlation energy calculated via eq 1. This indicates a weak coupling between long-range and short-range correlation effects, supporting a multiscale approach that aims for the TDL while neglecting the short-range.

**Figure 1:**
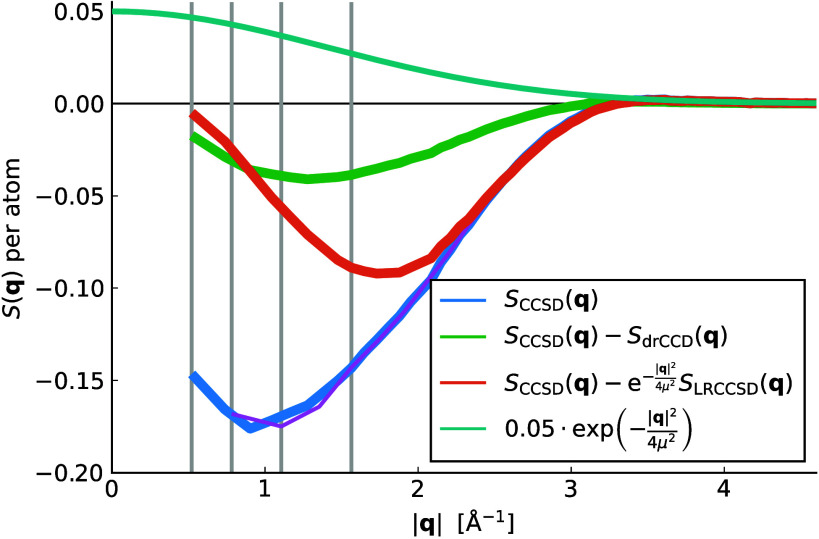
Transition structure factors and differences of structure factors in reciprocal space of metallic aluminum using finite supercells of 108 atoms. The thin purple line shows *S*_CCSD_(***q***) for a supercell of 32 atoms. The structure factors are spherically averaged; hence the area under each curve provides an estimate of the corresponding correlation energy. The four gray vertical lines indicate the smallest |***q***| vector for supercells with 108, 32, 16, and 4 atoms (left to right). Also the exponential damping function for the long-range potential is shown.

Additionally, the long-range potential offers a significant computational advantage by enabling a substantial reduction in the basis-set size, thereby greatly decreasing the computational workload. Consistent with previous observations, a much smaller basis-set can be used to reach the CBS.^42−45^Figure 2 shows the convergence rate for the correlation energy by using the long-range potential. This is because the electron–electron cusp is less sharp for a long-range potential compared to a Coulomb potential.^46^ Thus, we can systematically control the space of low-energy excitations required to accurately capture the long-range contributions to the ground-state energy. Note that the convergence rate to the CBS depends on the choice of μ, converging faster/slower for smaller/larger μ. In our setting, only about 3 unoccupied orbitals per occupied orbital are necessary to achieve an accuracy well below 5 meV per atom for the total energy. Such an accuracy is impossible to attain with current computational resources for total correlation energies using the Coulomb potential. Typically, around 20 or more optimized basis functions (such as natural orbitals^20,41^ or correlation consistent Gaussians^47,48^) per occupied orbital—in combination with effective correction schemes like the explicitly correlated F12 method^49^—are required, even for energy differences that benefit from significant error cancellation, to reach chemical accuracy (1 kcal/mol or about 43 meV) for observables like atomization or reaction energies.^21^

**Figure 2:**
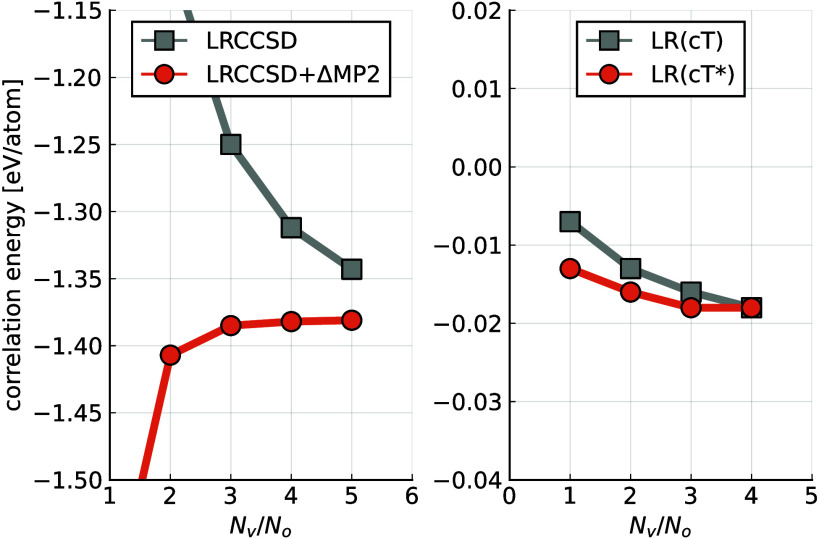
Rapid convergence of the total LRCCSD (left) and LR(cT) (right) correlation energy of metallic aluminum with respect to the number of basis funcitions per occupied orbitals, *N*_*v*_/*N*_*o*_. The basis functions are given by approximate natural orbitals.^41^ An even faster convergence can be achieved by adding a correction based on the complete basis-set limit of the MP2 level. Details on ΔMP2 and LR(cT*) are provided in the SI.^40^ In this work we chose *N*_*v*_/*N*_*o*_ = 3.

To estimate the reduction in computational cost, note that reducing the basis-set size by a factor of *x* decreases the memory footprint by a factor of *x*^2^ and the computation time by a factor of *x*^4^ at the CCSD level. For higher orders of CC theory, the savings are even more pronounced. A comparison of the computation time of both CC and LRCC calculations in dependence of the system size is provided in the SI.^40^

Based on these observations, we introduce a finite-size correction scheme to approximate the TDL for real metals modeled by finite supercells. The main idea is to shift the task of reaching the TDL of the CC ground-state energy to the less computationally demanding long-range contribution using the following equation,
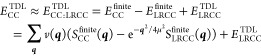
5The TDL in *E*_LRCC_^TDL^ can be estimated using existing techniques, such as cell size extrapolations or structure factor interpolations, but with greatly reduced computational cost. Computational details used for the following results can be found in the SI.^40^

Figure 3 illustrates the performance of this finite-size correction for the total CCSD(cT) energy of metallic aluminum, denoted as CCSD(cT):LRCCSD(cT). The energy difference between CCSD(cT) and LRCCSD(cT) saturates quickly with increasing cell size, making this correction scheme more effective than the one based on the structure factor interpolation,^32^ denoted as CCSD(cT)+FS. It must be fairly noted that the used implementation of the FS correction is not fully warranted for metallic systems, as it assumes a quadratic behavior of the structure factor around ***q*** = 0. This is the correct behavior for insulating systems, leading to a finite-size error scaling of *N*^–1^. Nevertheless, it has been shown that metals require a linear interpolation of the structure factor around small transition vectors, implying a *N*^–2/3^ behavior,^37^ where *N* is the number of atoms in the supercell.

**Figure 3:**
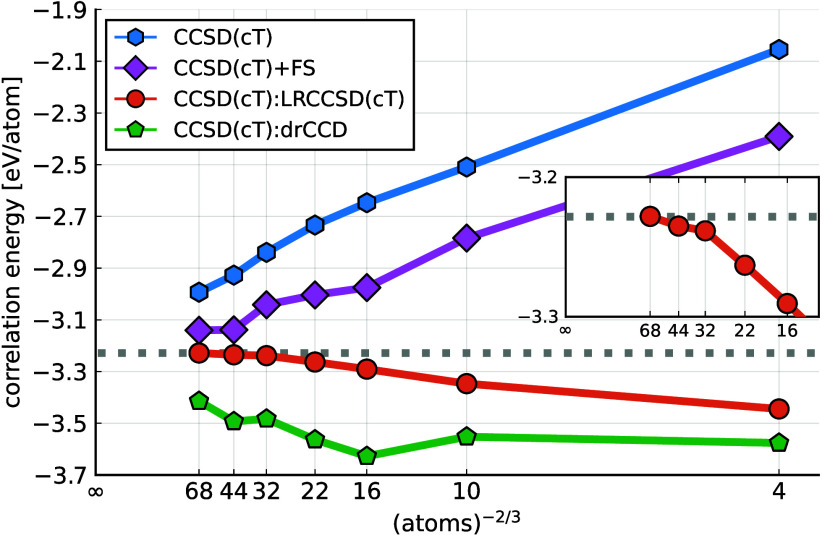
Approaching the total correlation energy per atom of metallic aluminum at the level of CCSD(cT) using different finite-size corrections schemes. The correction schemes are introduced in the main text. The horizontal axis corresponds to (number of atoms)^−2/3^, with labeled values corresponding to the number of atoms in the supercell. The dashed line shows the extrapolated CCSD(cT) energy.

Since the (cT) contribution is evaluated in a single-shot calculation, the finite-size effects can be corrected separately in the sense of CCSD(cT):LRCCSD(cT) = CCSD:LRCCSD + (cT):LR(cT). The separate convergence is documented in the SI(40) and shows that both the total correlation energy and the finite-size error are dominated by the CCSD contribution. While the CCSD contribution to the correlation energy is on the order of −3 eV, the (cT) contribution is on the order of −0.1 eV. The SI also shows that the long-range based correction outperforms the extrapolation technique at the level of CCSD.

Another advantage of the proposed correction scheme based on the long-range potential is that it accounts for the so-called minimum drifting of the structure factor, which was already described by Weiler et al.^50^ The drifting of the characteristic minimum of the structure factor can be observed in Figure 1, when comparing the CCSD structure factor of the 108-atom cell (blue) with 32-atom cell (thin purple line). The minimum shifts to the left for increasing cell sizes (i.e., finer ***q*** grids). Weiler et al. suggest that the drifting is due to relaxations of the CC amplitudes from eq 3 as the grid is refined. The CC:LRCC method avoids this issue, as the effects from the drifting can be assumed to cancel out in the difference .

Additionally, a correction based on the direct-ring coupled cluster doubles (drCCD) theory,^12,51^ denoted as CCSD(cT):drCCD, is considered. It is equivalently defined via eq 5 by replacing LRCC with drCCD. This correction draws from our previous works, where we showed that the long-range contributions of fragment-based applications of CCSD(T) theory can be effectively approximated with ring-like terms of CCSD for materials with a gap.^52,53^ However, as apparent from Figures 1 and 3 this does not seem to apply for the total correlation energy of metals. The difference of the structure factors of CCSD and drCCD exhibits a slower decay to zero for decreasing |***q***| (increasing cell sizes) as compared to the difference between CCSD and LRCCSD.

*Surface Energy.* We now turn to the surface energies of aluminum and platinum. The surface of a material determines its properties for scientific and industrial applications. It is defined by
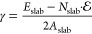
6where *E*_slab_, *N*_slab_, and *A*_slab_ represent the total energy, the number of atoms, and the surface area of the slab model, respectively. Here,  denotes the energy per atom of the bulk material. The factor of 2 in the denominator accounts for the two surfaces of the slab model. The technique to extract the surface energy from slab calculations largely follows the work by Fiorentini et al.^54^ and is described in the SI.^40^ The presented finite-size correction scheme enables us to study the convergence of systematically improved wave function methods by increasing the CC excitation orders—HF, CCSD, and CCSD(cT)—for surface energies in the TDL. The results are shown in Figure 4. While well-established DFT functionals, such as LDA,^55^ PBE,^56^ SCAN,^57^ as well as SCAN corrected by van der Waals (vdW) contributions (here rVV10, i.e., revised Vydrov–Van Voorhis 2010^58^), are at least close to the experimental values for Al(111), they severely underestimate the surface energy of Pt(111). In both cases, the systematically improved wave function methods show smooth convergence, with only minor corrections arising from contributions beyond CCSD. This suggests that surface energies converge rapidly with respect to the excitation order in CC theory. Notably, this outcome was achieved by employing a well-defined method to capture triple excitation effects, here (cT), which does not suffer from an infrared catastrophe as the perturbative triple approach (T), does. For both materials, CCSD(cT) demonstrates excellent agreement with the experimental values. In units of J/m^2^ we find surface energies of 1.17 for aluminum and 2.65 for platinum using CCSD(cT), compared to experimental values^10^ of 1.14 ± 0.20 and 2.49 ± 0.26, respectively. The residual numerical uncertainty in the computed CCSD(cT) estimates is determined in SI to be less than 0.1 J/m^2^. It is important to note that the available experimental results are relatively old and extrapolated to *T* = 0 K from surface tension measurements of the liquid phase, and specific terminations such as (111) are not directly accessible. Despite these limitations, the experimental results provide valuable, albeit uncertain, estimates for the low-energy faces of bulk crystals which are frequently referenced in notable studies.^8−11,59^ Additional deviations from experimental values may arise from effects not considered in this work, including contributions from frozen core electrons, relativistic effects of the valence electrons, and ionic relaxation of the slabs. Previous DFT studies, however, have shown that ionic relaxation effects are very small.^8^

**Figure 4:**
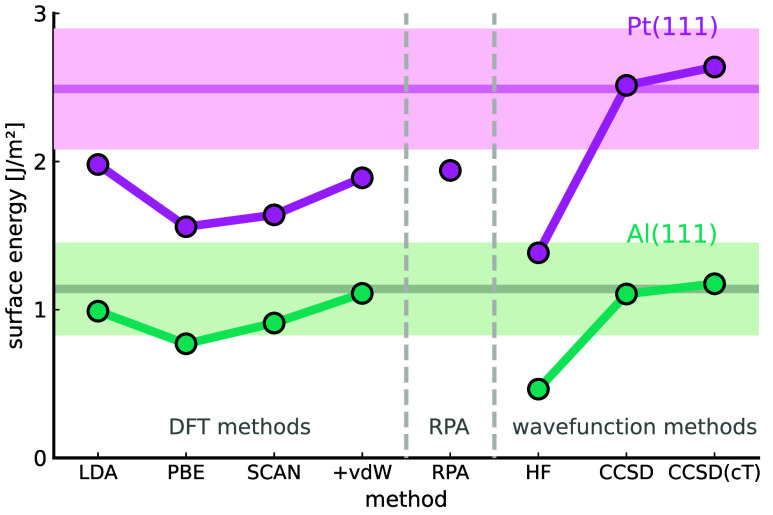
Surface energy of aluminum and platinum in the (111) termination from various methods. The experimental results (horizontal lines) and their uncertainties (lighter area), as well as the values of the different DFT functionals are taken from ref^10^ Here + vdW denotes SCAN+vdW as described in the text. The RPA result is taken from ref^9^

Notably, the finite-size correction for the CC correlation contribution to the surface energy in the *xy* direction (parallel to the surface) is virtually zero for the 2 × 2 slab of Al(111). This is dicussed in the SI.^40^ At first glance, this might seem contradictory to reports that show slower convergence in the *xy* direction.^60^ However, the separated treatment of electrostatic HF and correlation contributions to the surface energy reveal that the correlation contribution converges quickly with respect to the *xy* dimension of the slab. Twist-averaging plays an important role in this, as it smooths the convergence compared with the more erratic convergence observed with **Γ**-centered meshes for the BZ sampling. Thus, long-range effects in the *z* direction (normal to the surface) dominate the correlation part of the surface energy. This outcome, revealed through our novel finite-size correction scheme, underscores its ability and would have been difficult to detect otherwise. Details can be found in the SI.^40^

Our investigation also highlights a limitation of the finite-size correction based on the structure factor interpolation, denoted as CCSD(cT)+FS. This scheme predicts a CCSD(cT) result of 0.5 J/m^2^ for the surface energy of aluminum and 2.4 J/m^2^ for platinum, considering 2 × 2 surface slabs. The error introduced by this correction for aluminum arises from the interpolation of the structure factor in the *xy* direction. As illustrated in the SI,^40^ the smallest ***q*** vectors in the *xy* direction do not reach the characteristic minimum of the structure factor, leading to inaccurate finite-size corrections of the +FS method when interpolating down to ***q*** = 0.

## Conclusion

We introduce a novel finite-size correction scheme to enable coupled cluster theory for highly accurate materials modeling of metals. Using the example of metal surface energies, which are highly relevant due to their wide range of applications, we demonstrated that this observable can be reliably reproduced with high precision for the first time.

Furthermore, we employed a recently published methodology for treating approximate triple correlation effects, denoted as CCSD(cT). Our results add further evidence that this approximation is both accurate and practical for applications in metallic solids.

The proposed workflow to systematically converge CC calculations for metals can be considerably accelerated even further, when combined with structure factor interpolation techniques and *the shortcut to the TDL* proposed in ref (6) which allows the stochastic twist-averaging to be bypassed. Additionally, the weak coupling of different length scales in ***q*** can serve as a foundation for designing novel, reduced-cost algorithms.

This breakthrough paves the way for more efficient and more confident utilization of coupled cluster theory in materials science, including metals, necessary for research areas such as the rational design of heterogeneous catalysts, the development of new functional materials, and the provision of highly accurate benchmark results for machine learning techniques.

## Supporting Information Available

Details on the computational workflow, technique to calculate the surface energy, and computational timings (PDF)
