# Supplementary material for: Ground States for Metals from Converged Coupled Cluster Calculations
Source: J Phys Chem Lett. 2024 Dec 18;16(1):17–23. doi: 10.1021/acs.jpclett.4c03134 (PMC11726802; doi:10.1021/acs.jpclett.4c03134)
Supplement: Supplementary file 1 — jz4c03134_si_001.pdf [file jz4c03134_si_001.pdf]

Supplementary information for: Ground-States for Metals from Converged Coupled Cluster Calculations

Tobias Schäfer*

Institute for Theoretical Physics, TU Wien,

Wiedner Hauptstraße 8-10/136, A-1040 Vienna, Austria

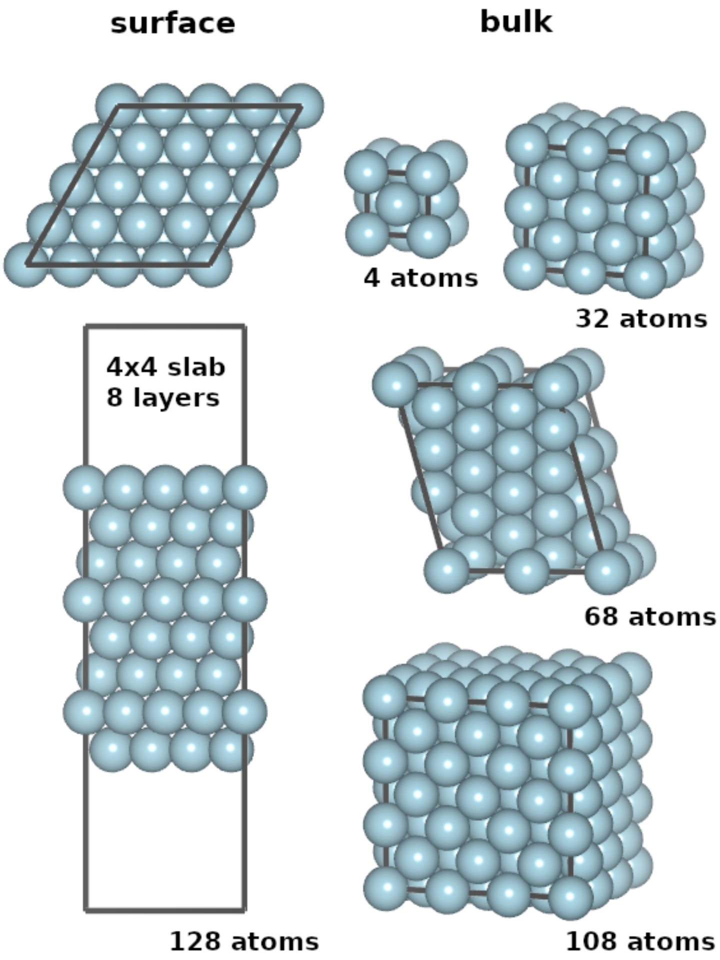

FIG. 1. Selected models used in this work. Surface models are shown on the left, bulk models on the right. A vacuum of 10 Å is chosen for the surface slabs. A lattice constant of 4.018 Å is considered . The lattice vectors are shown as dark gray lines. Aluminum atoms are represented as space-filling blue balls.

COMPUTATIONAL DETAILS AND WORKFLOW

All Hartree-Fock (HF) calculations are performed with the Vienna Ab-initio Simulation Package (VASP) [1–3] using a plane-wave cutoff of `ENCUT`=300 eV. The following pseudopotentials (`POTCAR` files) using the projector augmented wave (PAW) method [4] are employed: `PAW_PBE Al_GW` for aluminum providing 3 valence electrons and `PAW_PBE Pt_GW` for platinum providing 10 valence electrons. The coupled cluster (CC) calculations are performed with the CC4S code based on the interface from VASP [5]. For the surface slabs, a recently published

sampling technique for the Coulomb potential was used in order to achieve converged HF and CC iterations in strongly anisotropic simulation cells [6]. This sampling technique lets us use a 10 Å vacuum for the slab models, effectively eliminating interactions with periodic images in the z direction due to the distance.

Workflow

In the following, we describe the general workflow for CC calculations, performed for the main manuscript. (VASP) [1–3] and the `Cc4s` [7] code. All correlation calculations are performed based on a single k-point sampling of the Brillouin zone (BZ) using increasingly large supercells. A selection of the used models for aluminum is visualized in Fig. 1. The following procedure is repeated for randomly chosen k-points, a Monte-Carlo technique called twist-averaging [8, 9].

1. The HF ground state is calculated. Both the occupied as well as all unoccupied orbitals and orbital energies are stored.

2. Approximate natural orbitals at the MP2 level are computed as described in Ref. [10]. Natural orbitals are the eigenvectors of the one-electron reduced density matrix, with their corresponding eigenvalues referred to as occupation numbers. Arranged by their occupation numbers, we truncated and recanonicalized the natural orbital basis by selecting a ratio N_v/N_o, where N_o represents the number of occupied orbitals in the system and N_v represents the number of chosen natural orbitals. This basis of natural orbitals facilitates a much quicker convergence of the correlation energy with respect to N_v.

3. The MP2 energy is calculated in the CBS limit using the natural orbitals with $N_v/N_o = 100$. This is necessary for basis set correction schemes to estimate the CBS limit of the CCSD and (cT) energies. The basis set correction scheme (called focal point correction) is described in Ref. [11]. For (cT) we use the (cT*) method in analogy to (T*) [12] in order to estimate the complete basis set limit (CBS). The (cT*) estimate is obtained by rescaling (cT) with the relation between the small basis result of MP2 and the CBS of MP2.

   In the case of the long-range potential $N_v/N_o = 16$ is chosen.

4. For the coupled cluster calculations, a smaller basis, defined by $N_v/N_o = 8$, is selected. Basis set convergence is discussed below. All Coulomb integrals, V^{pq}_{sr}, required by coupled cluster theory are calculated using the expression:

$$V^{pq}_{sr} = \sum_{F=1}^{N_F} \Gamma^{*pF}_{s} \Gamma^{q}_{rF}, \tag{1}$$

where p, q, r, s are indices for occupied or virtual orbitals. F denotes auxiliary basis functions obtained via singular value decomposition as described in Ref. [13]. Due to the significant vacuum in the surface slabs, the auxiliary basis set size can be considerably reduced without sacrificing the precision of the computed correlation energies. The correlation energies are converged to within meV relative to the size of the optimized auxiliary basis set.

5. The final coupled cluster calculations at various levels (drCCD, CCSD, and CCSD(cT)) are executed using the high-performance code cc4s.

Complete basis set limit of the long-range coupled cluster results

As shown in the main manuscript the rapid convergence of the long-range coupled cluster energies with respect to the basis-set size N_v/N_o can be accelerated by adding a correction based on the MP2 energy via

$$\text{LRCC} + \Delta\text{MP2} = \text{LRCC(small basis)} - \text{LRMP2(small basis)} + \text{LRMP2(CBS)}\ . \tag{2}$$

We also define

$$\text{LR(cT*)} = \text{LR(cT)(small basis)} \cdot \frac{\text{LRMP2(CBS)}}{\text{LRMP2(small basis)}}\ . \tag{3}$$

In this work we used a fix setting of $N_v/N_o = 3$ for CC and $N_v/N_o = 16$ for MP2.

CALCULATION OF THE SURFACE ENERGY

Several procedures to calculate surface energies of periodic slabs were proposed in the literature. In this work, we employ the fitting technique [14], where the surface energy γ is found by fitting the ground-state energies $E(N_{\text{layer}})$ against the number layers N_{layer} through

$$E(N_{\text{layer}}) = 2 \cdot A \cdot \gamma + N_{\text{layer}} \cdot E_b\ . \tag{4}$$

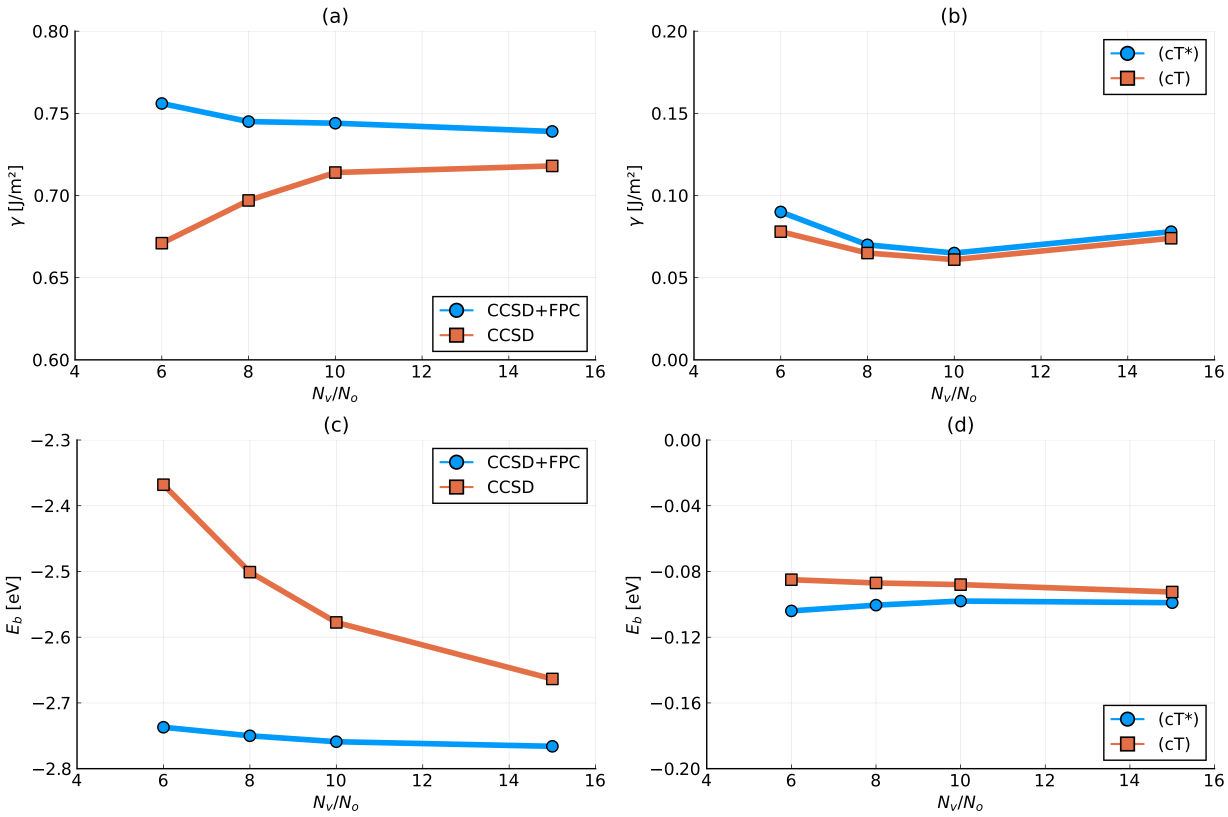

FIG. 2. Basis-set convergence of the fit parameters γ and E_b of the fit $E(N_{\text{layer}}) = 2A\gamma + N_{\text{layer}}E_b$ for aluminum. A single twist angel was used. Only $N_{\text{layer}} = 4, 6$ was considered in order to directly capture the basis-set effect without averaging effects from multiple layers.

Here, A is the surface area of the slab and the other fitting parameter, E_b, can be interpreted as an estimate for the bulk energy.

For aluminum we considered $N_{\text{layer}} = 2, 4, 6, 8$. As one aluminum atom provides an odd number of electrons, i.e. 3, in our setting, only even numbers of layers are chosen to ensure an even number of electrons in the 3×3 slab. For platinum we considered $N_{\text{layer}} = 2, 3, 4, 5$, as one platinum atom provides an even number of electrons, i.e. 10. Note that it was already shown in Ref. [15] that the Pt(111) surface energy converges fast with respect to the number of layers using the fitting technique.

The HF contribution for both Al and Pt was calculated using a $n \times n \times 1$ sampling of the BZ with $n = 12$ and additionally averaging over stochastic twist angles, carefully checking for convergence with n and the number of twist angles.

Complete basis set limit of the surface energy

The basis-set dependence of the surface energy of aluminum is shown in Fig. 2. The highly effective focal point correction [16] allows us to use $N_v/N_o = 8$ to achieve CCSD surface energies with a remaining uncertainty of less than $0.05\,\mathrm{J/m^2}$. Note that even $N_v/N_o = 15$ does not provide the same accuracy without the basis-set correction. For the triples contributions, (cT), we find a much weaker basis-set dependence. The choice of $N_v/N_o = 8$ introduces an even smaller error, especially when the (cT*) correction is used, as explained in Eq. (3).

FINITE-SIZE CORRECTION SCHEME APPLIED TO BULK AND SURFACE

As defined in the main manuscript, the finite-size correction scheme shifts the task to reach the thermodynamic limit (TDL) to the long-range coupled cluster (LRCC) part as

$$E_{\mathrm{CC}}^{\mathrm{TDL}} \approx E_{\mathrm{CC:LRCC}}^{\mathrm{TDL}} = E_{\mathrm{CC}}^{\mathrm{finite}} - E_{\mathrm{LRCC}}^{\mathrm{finite}} + E_{\mathrm{LRCC}}^{\mathrm{TDL}}\,. \tag{5}$$

In this work we estimate $E_{\mathrm{LRCC}}^{\mathrm{TDL}}$ via cell size extrapolations based on cells with 32, 44, 68 and 108 atoms. For the bulk an extrapolation low of $N^{-2/3}$ is assumed, where N is the number of atoms per cell. In Fig. 3 we show the convergence of the individual contributions to the correlation energy of bulk aluminum. Additionally, the convergence of the extrapolation technique is visualized.

Considering the surface, Fig. 4 shows the correlation contribution from CCSD(cT) and LRCCSD(cT) to the surface energy using slabs growing in the xy direction in dependence of the used stochastic shifts for the twist-averaging technique. The stochastic noise of the twist-averaging leaves an uncertainty of about $0.1\,\mathrm{J/m^2}$ for the 4×4 slab. For all other systems the uncertainty is smaller. Within this error bar, the correlation contribution is already converged at the 2×2 slabs.

To hedge this surprising result, we also considered the change of the direct-ring coupled cluster doubles (drCCD) surface energy from 2×2 slabs to 3×3 slabs using the full Coulomb potential. This is possible as drCCD is computationally less demanding than CCSD or CCSD(cT) (CCSD(cT)) calculations. Clearly, the drCCD correlation contribution to the surface energy agree for both the 2×2 slab and the 3×3 slab within an error of less that $0.05\,\mathrm{J/m^2}$. In other words, the correlation contribution to the surface energy does indeed

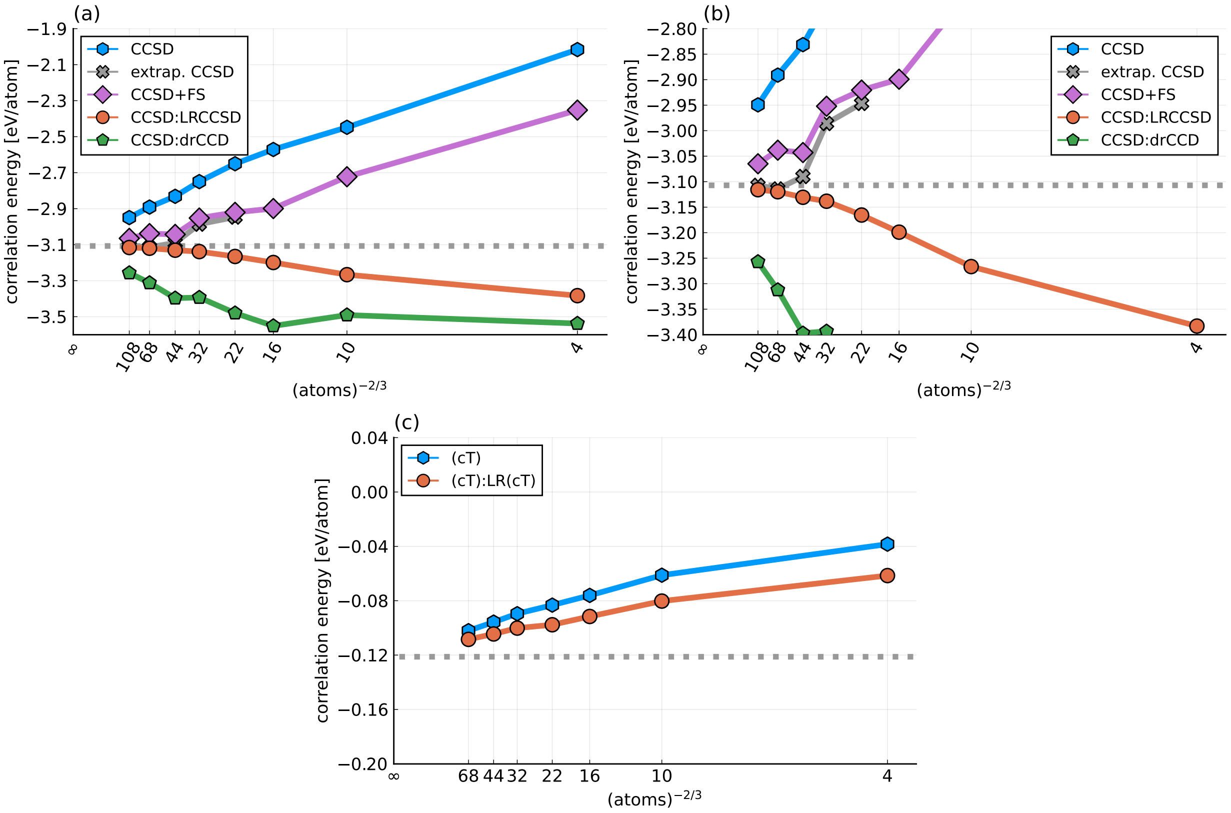

FIG. 3. Approaching the total correlation energy per atom of metallic aluminum at the level of (a)&(b) CCSD and (c) (cT) using different finite-size corrections schemes. The correction schemes are introduced in the main text. The finite-size of the periodic model is indicated by the number of atoms on the horizontal axes. The extrapolation is performed using the largest four data points and is denoted as "extrap. CCSD". The best extrapolated CCSD / (cT) energy is shown as a dashed line.

only have a very weak dependence on the xy direction. To keep the computationally cost as low as possible, we neglected a basis-set correction for the drCCD results of this test.

Turning to the finite-size correction based on the structure factor interpolation [17], we observe a massive underestimation of surface energies. As shown in Fig. 5, the 2×2 structure factor does not reach its characteristic minimum for vectors in the xy direction. Hence, the interpolation introduces severe numerical errors, leading to the wrong CCSD(cT)+FS estimates for the surface energy, mentioned in the main manuscript

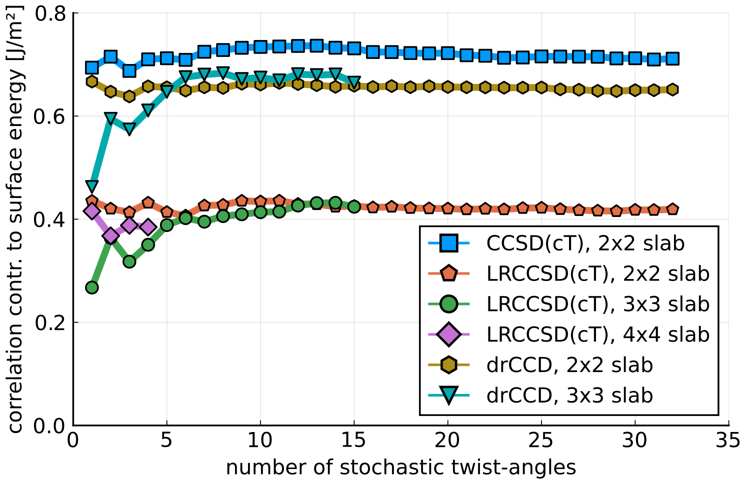

FIG. 4. Dependence of the correlation contribution to the surface energy of Al(111) with respect to the number of stochastic shifts for the twist-averaging. Only for consistency we note that the CCSD(cT) as well as LRCCSD(cT) results are basis-set corrected while the drCCD results are not.

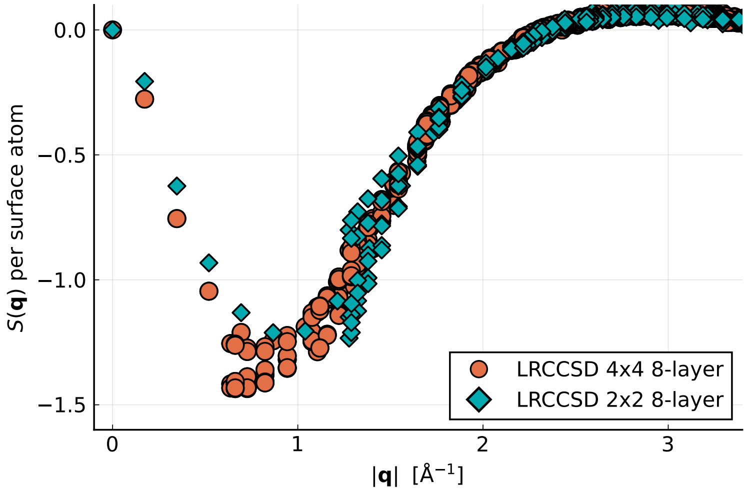

FIG. 5. LRCCD structure factors of $n \times n$ aluminum surface slabs with 8 layers and $n = 2, 4$ using a single twist. Note that that the considered supercells are strongly anisotropic.

TIMINGS AND COMPUTER ARCHITECTURE

Figure 6 shows the CC computation time for 8-layer aluminum slabs. Up to 32 nodes with 128 cores each were used for the largest calculations. The nodes are equipped with AMD EPYC 7713 CPUs and 512 GB of main memory. They are provided by the Vienna Scientific Cluster (VSC-5).

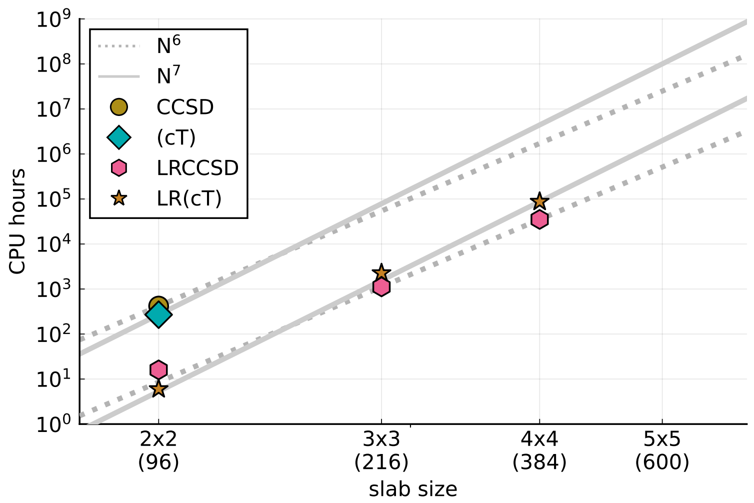

FIG. 6. Log-log plot of the computation time for Aluminum slabs with 8 layers. Both CCSD timings are given per 20 iterations, providing a rough estimate for the total required. The number of electrons in the slab model is given in brackets.

---

* tobias.schaefer@tuwien.ac.at

[1] G. Kresse and J. Hafner, Ab initio molecular dynamics for liquid metals, Physical Review B **47**, 558 (1993).

[2] G. Kresse and J. Furthmüller, Efficient iterative schemes for ab initio total-energy calculations using a plane-wave basis set, Physical Review B **54**, 11169 (1996).

[3] G. Kresse and J. Furthmüller, Efficiency of ab-initio total energy calculations for metals and semiconductors using a plane-wave basis set, Computational Materials Science **6**, 15 (1996).

[4] P. E. Blöchl, Projector augmented-wave method, Physical Review B **50**, 17953 (1994).

[5] cc4s, `https://manuals.cc4s.org`.

[6] T. Schäfer, W. Z. V. Benschoten, J. J. Shepherd, and A. Grüneis, Sampling the reciprocal coulomb potential in finite anisotropic cells, Journal of Chemical Physics **160**, 51101 (2024).

[7] T. Gruber, K. Liao, T. Tsatsoulis, F. Hummel, and A. Grüneis, Applying the coupled-cluster ansatz to solids and surfaces in the thermodynamic limit, Physical Review X **8**, 021043 (2018).

[8] C. Lin, F. H. Zong, and D. M. Ceperley, Twist-averaged boundary conditions in continuum quantum monte carlo algorithms, Physical Review E **64**, 016702 (2001).

[9] T. N. Mihm, L. Weiler, and J. J. Shepherd, How the exchange energy can affect the power laws used to extrapolate the coupled cluster correlation energy to the thermodynamic limit, Journal of Chemical Theory and Computation **19**, 1686 (2023).

[10] A. Grüneis, G. H. Booth, M. Marsman, J. Spencer, A. Alavi, and G. Kresse, Natural orbitals for wave function based correlated calculations using a plane wave basis set, Journal of Chemical Theory and Computation **7**, 2780 (2011).

[11] A. Irmler, A. Gallo, and A. Grüneis, Focal-point approach with pair-specific cusp correction for coupled-cluster theory, The Journal of Chemical Physics **154**, 234103 (2021).

[12] G. Knizia, T. B. Adler, and H. J. Werner, Simplified ccsd(t)-f12 methods: Theory and benchmarks, Journal of Chemical Physics **130**, 10.1063/1.3054300/908511 (2009).

[13] F. Hummel, T. Tsatsoulis, and A. Grüneis, Low rank factorization of the coulomb integrals for periodic coupled cluster theory, The Journal of chemical physics **146**, 124105 (2017).

[14] V. Fiorentini and M. Methfessel, Extracting convergent surface energies from slab calculations, Journal of Physics: Condensed Matter **8**, 6525 (1996).

[15] N. E. Singh-Miller and N. Marzari, Surface energies, work functions, and surface relaxations of low-index metallic surfaces from first principles, Physical Review B **80**, 235407 (2009).

[16] A. Irmler, A. Gallo, F. Hummel, and A. Grüneis, Duality of ring and ladder diagrams and its importance for many-electron perturbation theories, Physical Review Letters **123**, 156401 (2019).

[17] K. Liao and A. Grüneis, Communication: Finite size correction in periodic coupled cluster theory calculations of solids, The Journal of Chemical Physics **145**, 141102 (2016).
